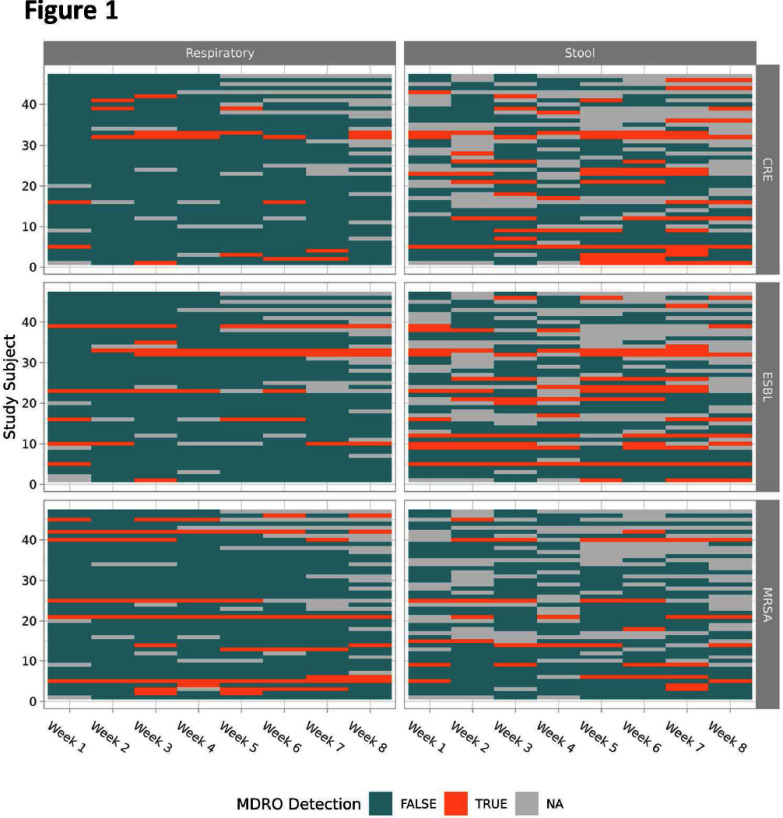# High prevalence of multidrug resistant organisms in a pediatric post-acute care unit

**DOI:** 10.1017/ash.2025.359

**Published:** 2025-09-24

**Authors:** Kathleen Chiotos, Jeffrey Gerber, Teresa Arroyo, Dylan Tapper, Alexa Patel, Laura Cowden, Pam Tolomeo, Ebbing Lautenbach, Susmita Tarafdar, Richard Lin, Brendan Kelly

**Affiliations:** 1 Childrens Hospital of Philadelphia; 2 University of Pennsylvania School of Medicine; 3 University of Pennsylvania; 4 University of Pennsylvania Perelman School of Medicine; 5 Perelman School of Medicine, University of Pennsylvania; 6 AARC; 7 The Children’s Hospital of Philadelphia; 3 University of Pennsylvania

## Abstract

**Background:** The prevalence of multidrug-resistant organisms (MDROs) in the post-acute care setting is well-documented in adults. Few studies have investigated the prevalence in children. **Methods:** We performed a prospective, single-center study including children with tracheostomy tubes age 2 months to 17 years admitted to a 24-bed post-acute care unit within a quaternary care children’s hospital. Index respiratory and stool specimens were obtained within two weeks of admission. Subsequent specimens were obtained weekly thereafter for up to eight weeks. MDROs were identified using methicillin-resistant Staphylococcus aureus (MRSA), extended-spectrum beta-lactamase Enterobacterales (ESBL-E), and carbapenem-resistant Enterobacterales (CRE) selective media (CHROMagar, Hardy Diagnostics). ESBL-E and CRE colonies were additionally plated onto MacConkey agar and only lactose fermenting organisms were considered positive. Index MDRO status was defined using week one samples; if not available, week two results were substituted. New MDRO acquisition was defined as a negative index MDRO culture with a subsequent positive culture. **Results:** A total of 47 children were enrolled. Median age was 9 months (interquartile range [IQR], 5-31 months) and median hospital length of stay prior to post-acute care admission was 89 days (IQR 27, 158). The most common pre-existing medical conditions were congenital heart disease (19, 40%), severe neurologic impairment (19, 40%), and prematurity **Conclusion:** MDROs are common in children hospitalized in the post-acute care unit. Nearly half of this cohort acquired CRE following admission, highlighting the need for strict infection prevention and control measures and tailored empiric antibiotic strategies.

**Figure:**